# Exposure of haptic of posterior chamber intraocular lens after sutureless intrascleral fixation

**DOI:** 10.1186/s12886-015-0102-3

**Published:** 2015-08-14

**Authors:** Yoshitsugu Matsui, Hisashi Matsubara, Tsukasa Hanemoto, Mineo Kondo

**Affiliations:** Department of Ophthalmology, Mie University Graduate School of Medicine, 2-174 Edobashi, Tsu, Mie 514-8507 Japan; Kozawa Eye Hospital, Ibaragi, Japan

## Abstract

**Background:**

A technique of sutureless intrascleral fixation of an intraocular lens (IOL) in an eye that lacks a posterior capsular support has been reported. The advantage of this technique was that the suture-related complications did not develop. However, the long-term complications of a sutureless IOL implantation have not been reported.

**Case presentation:**

A 75-years-old man had a sutureless intrascleral fixation (Y-fixation) of an IOL 4 months before our examination. The nasal haptic became exposed and the temporal haptic was seen in the subconjunctiva. The tilted IOL was removed and replaced by a posterior chamber IOL that was sutured to the sclera. At the 6 months examination, the eye was quiet and the IOL was stable.

**Conclusion:**

We suggest that the exposure of the nasal haptic of an IOL that was implanted by sutureless intrascleral fixation (Y-fixation) was due to poor surgical technique and/or the erosion of a fragile sclera. Thus, eyes should be carefully and frequently monitored after sutureless intrascleral posterior chamber IOL implantation.

## Background

An implantation of an intraocular lens (IOL) in eyes that lack a posterior capsular support has been accomplished with an iris-fixed IOL, an anterior chamber IOL, or a transscleral IOL fixed by sutures passed through the ciliary sulcus or pars plana. In 2006, Gabor introduced a transscleral needle fixation of an IOL [1], and in 2007, Agarwal introduced the glued IOL technique in which the intrascleral haptic of a posterior chamber IOL is glued to the sclera [2]. In 2012, Ohta introduced the Y-fixation technique that fixed an IOL without large lamellar scleral flaps and fibrin glue [3].

The advantage of these techniques is that it avoided suturing and thus the suture-related complications, and it required no specially designed haptics. The sutureless intrascleral fixation technique allowed an exact centration of the IOL and provided axial stability [4, 5]. However, there are only a few long-term follow-up studies on the complications of these techniques. We report a case in which the IOL haptic became exposed three months after the IOL was implanted with the Y-fixation technique.

## Case presentation

A 75-year-old man was referred to our hospital with an exposed IOL haptic. He had undergone lensectomy for a cataract in his left eye 30-years earlier but no surgical details were available. He then had an extracapsular IOL implantation 20-years earlier and the out-of-the-bag IOL dislocated 1-year ago. He then had another IOL (NX-70, Santen, Japan) implanted with the Y-fixation technique.

Three months postoperatively, the nasal haptic of the IOL haptic was found to be exposed and a self-scleral patch was grafted to cover the haptic at the previous hospital. However, the haptic became exposed again 1 month after the surgery, and he was referred to our hospital with a 1-month history of exposed IOL haptic in his left eye.

**Fig. 1:**
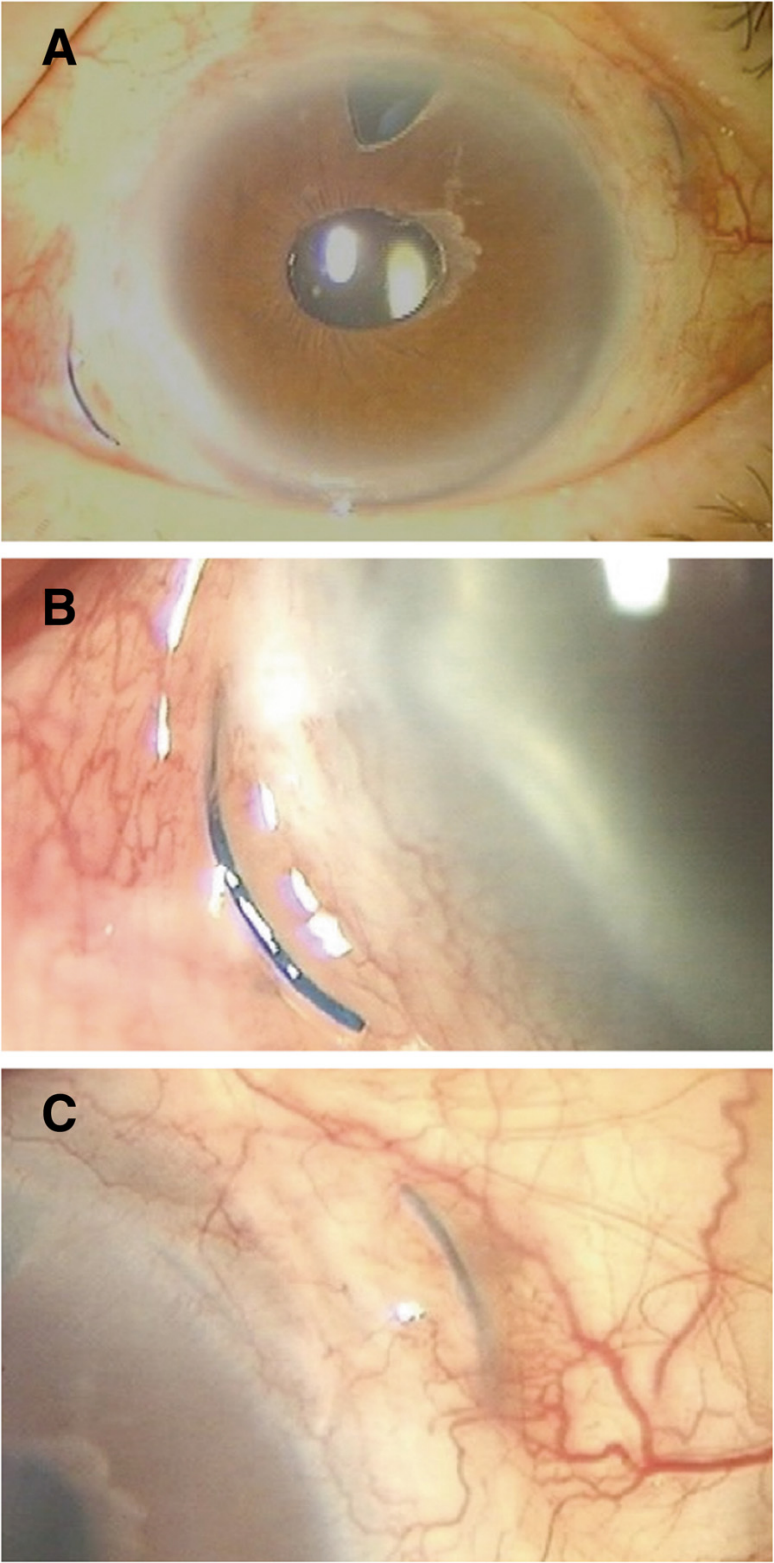
Photograph of the anterior segment of the eye showing an exposed haptic of an intraocular lens (IOL). **a** Preoperative view of the anterior segment. **b** The nasal haptic of an intraocular lens (IOL) is visible. **c** The temporal haptic can be seen in the subconjunctiva

Our examination showed that his decimal best-corrected visual acuity (BCVA) was 0.5 with a tilted IOL. The nasal IOL haptic was exposed and the temporal haptic could be seen in the subconjunctiva (Fig. 1a-c). To correct this condition, the conjunctiva around the haptics was opened, and the both haptics exposed. The Y-shaped incision and scleral flap were not detected. The tilted IOL was removed and a posterior chamber IOL was implanted and sutured.

At the 6 months follow-up examination, the eye was stable and the BCVA was 1.0. The IOL was fixed and aligned.

### Discussion

As best we know, another case of haptic exposure after sutureless Y-fixation has not been published. There are several possible explanation for the exposure of the nasal haptic. One is that a scleral tunnel was not constructed correctly. This procedure requires the creation of the scleral tunnel parallel to the limbus at the branching point of a Y-shaped incision. The scleral tunnel must be thick enough to prevent the protrusion of the haptic. Kumar has reported that the depth of the tunnel can alter the degree of scleral erosion by the haptic, and a subconjunctival haptic exposure was seen in three eyes (1.4 %) with thin scleral flaps [6]. Ohta recommended a 24-gauge MVR knife be used to create the sclerotomy for the appropriate haptic fixation instead of a needle [7]. It is easy to create an incision having a uniform thickness sclera with this knife. Ohta also recommended a single 8–0 nylon suture should be used to fix the haptic to the scleral bed to prevent it from shifting after surgery. In our case, the single 8–0 nylon suture approach was used in previous surgery at another hospital, but the haptic became malpositioned after 3 month of the Y-fixation technique.

**Fig. 2:**
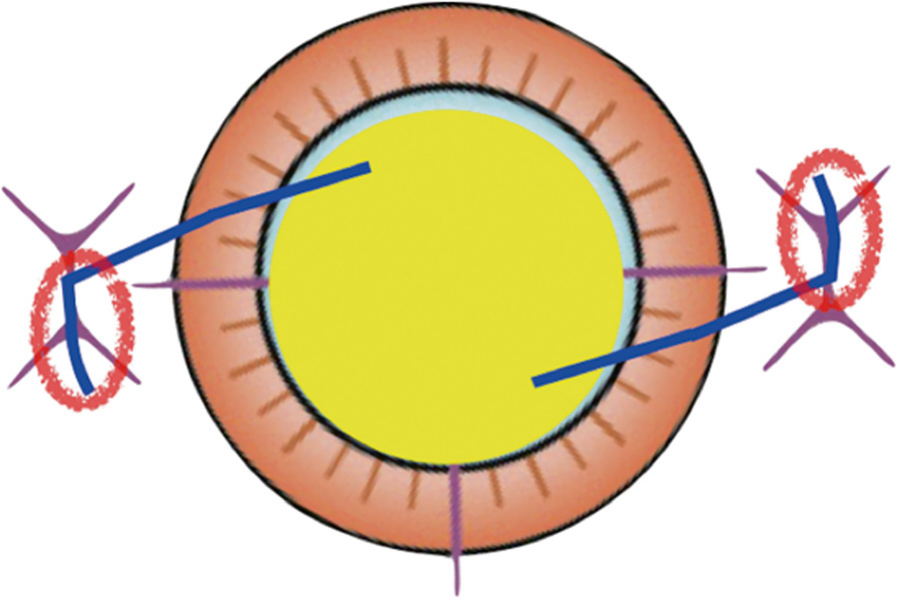
Diagram showing how a curved haptic can cause tension on the sclera when the IOL is inserted in a linear sclera tunnel. This may cause the torsion and erosion of the sclera (red circles)

A second factor for the haptics exposure is that an IOL specifically designed for sutureless intrascleral posterior chamber IOL implantation is not available. However, Gabor stated there is no need for special IOL designs for this method even though it is not known whether the structure of the sclera can be maintained for a long time. The average diameter of the cornea in Japanese is 11.5 to 12 mm, and the Y-shaped incision is made 2.0 mm from the limbus. Therefore, the full diameter becomes approximately 14 mm for the scleral fixation. However, an IOL is designed for intracapsular fixation, and the full length of an IOL with an optic diameter of 7.0 mm IOL is 13.5 mm in the bag. When this curved haptic is inserted in a linear sclera tunnel, an unnatural tension is placed on the sclera. This may cause torsion of the IOL and erosion of the sclera (Fig. 2). Kumar reviewed the complications in 486 eyes that had a single-piece PMMA IOL with an optic diameter of 6.5 mm implanted. The IOL haptic-related complication were; haptic displacement (4.1 %), haptic tip extrusion (0.8 %), subconjunctival haptic (0.4 %), and no exposed IOL haptic [8]. In addition, the incidence of exposed suture knots through the conjunctiva was 3.8 to 11 % in the late postoperative period of suture-assisted scleral fixated IOL implantations [9–12].

Both an exposed IOL haptic and exposed sutures are potential risks of endophthalmitis. However, long-term follow-up studies are lacking, and further studies comparing the structure of the sclera and the status of the sutures and haptic need to be conducted.

We recommend that the fragility of the sclera be estimated preoperatively. In our case, a self-scleral patch was grafted to cover the haptic 3 months after the Y-fixation technique surgery, but 1 month after the surgery the haptic was exposed again. This suggested that our patient had relatively fragile sclera. The patient did not have a medical history of inflammation such as scleritis, episcleritis, rheumatoid arthritis, and herpes zoster ophthalmicus, but had a history of two cataract surgeries. Many cases of sutureless intrascleral posterior chamber IOL implantation may be a second implantation surgery that had had scleral incisions. In addition, damage of the sclera may have occurred in the preoperatively from aggressive thermo-coagulation hemostasis of the sclera and insufficient controlling inflammation. Thus, when the Y-fixation technique is used, it is important to avoid damage to the sclera to maintain the integrity of the sclera for the implantation surgery.

## Conclusions

In conclusion, we have presented our findings in a case of an exposed haptic of an IOL after implantation with the sutureless Y-fixation technique. We recommend that the surgeon create a scleral tunnel correctly, and the sclera be carefully examined to determine if there are any signs of fragility before this procedure is used.

## Consent

The patient was fully informed about the examinations and provided written consent. Written consent was obtained from the patient for publication of this case report and any accompanying images. A copy of the written consent is available for review by the Editor.
